# Editorial: Advances in the treatment of chronic myeloid leukemia

**DOI:** 10.3389/fonc.2023.1166588

**Published:** 2023-03-09

**Authors:** Ahmet Emre Eşkazan, Chung H. Kok, David Yeung, Jignesh Dalal, Mario Tiribelli

**Affiliations:** ^1^ Division of Hematology, Department of Internal Medicine, Cerrahpaşa Faculty of Medicine, Istanbul University-Cerrahpaşa, Istanbul, Türkiye; ^2^ Precision Cancer Medicine Theme, South Australian Health & Medical Research Institute (SAHMRI), Adelaide, SA, Australia; ^3^ Adelaide Medical School, University of Adelaide, Adelaide, SA, Australia; ^4^ Centre for Cancer Biology, University of South Australia, Adelaide, SA, Australia; ^5^ Department of Haematology, Royal Adelaide Hospital and SA Pathology, Adelaide, SA, Australia; ^6^ Division of Hematology and Bone Marrow Transplant, Rainbow Babies and Children’s Hospital, OH, United States; ^7^ Case Western Reserve University, Cleveland, OH, United States; ^8^ Division of Hematology and Bone Marrow Transplant, Azienda Sanitaria Universitaria Friuli Centrale (ASUFC) – Udine Hospital, Udine, Italy; ^9^ Department of Medicine, University of Udine, Udine, Italy

**Keywords:** adverse event, arterial occlusive event, BCR::ABL1 transcript, COVID-19, quality of life, stem cell transplantation, treatment-free remission, tyrosine kinase inhibitor

The introduction of tyrosine kinase inhibitors (TKIs) has revolutionized the treatment of chronic myeloid leukemia (CML). Within the era of TKIs, many advances in the management of this disease have been achieved in a continuous manner. The Research Topic of the *Frontiers in Oncology* includes 6 papers on the management of CML, which will be summarized below.

The outcomes of TKI therapy in CML can be influenced by many disease- and patient-related factors including the *BCR::ABL1* transcript type (i.e., e13a2 and e14a2). In the literature there are conflicting results on the impact of transcript type on TKI responses and survival (1). Thus, Chen et al. performed a meta-analysis including 16 studies. With some limitations as stated by the authors, in this meta-analysis expression of e14a2 was found to be associated with faster, deeper, and more sustained molecular responses together with a survival benefit including overall survival (OS) when compared to e13a2. As treatment-free remission (TFR) is a desirable endpoint for patients with CML, this analysis also showed that having an e14a2 transcript could have a positive impact on achieving TFR.

Although multiple TKIs have proven effective for the treatment on CML, many patients can be affected by TKI-related toxicities. Whilst life-threatening events are thankfully uncommon, less severe, but more common side effects may have significant impact on quality of life (QoL). CML physicians should receive credit for minimizing the impact of these adverse events (AEs), through recognition of both commonly associated and idiosyncratic events, and the early diagnosis and optimal management these toxicities. To further aid this effort, Yoshifuji and Sasaki have comprehensively examined the characteristics and AEs profile of the 5 currently available ATP-competitive *BCR::ABL1* TKIs (imatinib, bosutinib, dasatinib, nilotinib, and ponatinib), together with the most recently approved agent targeting the ABL1 myristoyl pocket – asciminib. Several studies have now provided the basis for dose reduction to minimize toxicity yet preserving efficacy – most of them included in this review with expert commentary. Proactive dose optimization strategies will be increasingly important to personalize therapy, particularly in older patients who are at higher risk of toxicity.

As underlined by the review summarized above, dose reductions of TKIs can be performed in the presence of and/or to avoid potential TKI-associated AEs. There are not many studies evaluating TFR in patients receiving TKIs at lower than the recommended daily doses: to address this point, Iurlo et al. performed a multi-center study. Of a total number of 5.637 patients, 1.785 (31.7%) were receiving low-dose TKIs, and 64.4% of the hematologists involved believed that in patients receiving reduced doses of TKIs, a TFR can be attempted. One hundred and ninety-four patients attempted TFR, of which 98.4% had already achieved deep molecular response at the time of TFR. Following a median of approximately 30 months of follow-up, 71.1% patients were still in TFR, demonstrating that dose reduction can be performed successfully in a group of patients before attempting TFR, although more data should be accumulated to draw firmer conclusions.

Many TKIs are approved in the management of CML and choosing the appropriate TKI for individual patient based on treatment goals, age, comorbidities, and the AE profile of the available drugs. Although no significant difference in OS has been reported between imatinib and second-generation TKIs (2GTKIs), 2GTKIs were associated with increased risk of arterial occlusive events (AOEs) and venous thromboembolism (OR of 2.81 and 1.74, respectively) in retrospective studies (2).

In the present Research Topic, Sicuranza et al. reported result of prospective study including 186 CML patients (89 imatinib, 59 nilotinib, and 38 dasatinib) showing that a pro-inflammatory/prooxidative milieu developed during treatment with nilotinib compared with imatinib or dasatinib, as demonstrated by higher high‐sensitivity C‐reactive protein (hs‐CRP) and oxidized low density lipoprotein (oxLDL) levels and increased interleukin 6 (IL-6)/IL-10 and tumor necrosis factor alpha (TNF-α)/IL-10 ratios only in nilotinib cohort. After median follow‐up of 23.3 months from TKI start, 10/186 patients (5.4%) suffered an AOE and 5/10 (50%) AOEs occurred during nilotinib treatment despite a lower 10-year ‘Systematic Coronary Risk Evaluation’ (SCORE) and a lower median age in this subgroup. They demonstrated a progressive increase in oxLDL levels during nilotinib treatment, but not during imatinib or dasatinib.

Based on this data it was concluded that combination of inflammatory and oxidative mechanisms, that are closely related in atherogenesis and atherothrombotic complications, may eventually be responsible for nilotinib-associated endothelial activation leading to increased AOE. A longer follow-up is needed to further confirm the active role of nilotinib in AOE pathogenesis.

Pediatric CML is extremely rare, as the number of allogeneic hematopoietic stem cell transplantation (HSCT) performed in CML decreased considerably in the era of TKIs. In addition, data regarding the impact of HSCT on long-term health related QoL (HRQoL) in pediatric CML patients are lacking, that’s why the study by Schleicher et al. is quite important. The authors demonstrated the patient-reported outcomes (PROs) and HRQoL in 86 long-term survivors receiving an allograft prior to and after the introduction of TKIs. Late secondary malignancies and CML relapse were observed in 13% and 27% of the patients, respectively. The rate of chronic graft-versus-host disease (cGvHD) was 30%, with a negative impact on HRQoL, while cases without cGvHD had no differences in terms HRQoL when compared to controls.

In the last years, many studies on the impact of COVID-19 pandemic in patients with hematological neoplasms has been performed, with conflicting results. In CML, an Italian study reported an extremely low prevalence of infection (0.17%) (3). However, little is known on the impact of the pandemic on the management of patients and on CML outcomes.

In this Research Topic, Yılmaz et al. reported on cross-sectional study on 210 CML patients receiving regular outpatient care at a single institution in Turkey, comparing data in the year span before and after SARS-CoV2. More than 80% of patients were receiving imatinib, and 90% had achieved at least a major molecular response (MMR). Despite a significant reduction, compared to the previous year, both in median number of clinical visits (1 vs. 4) and molecular assessment (1 vs. 3) per patient, there was no reduction in TKI therapy adherence, that was higher in the pandemic year (88.8% vs. 78.1%) nor a detrimental effect on CML outcome. Only a minority of patients lost MMR (4/182), lost complete cytogenetic response (1/182) or progressed to blast phase (2/182); these number were like those observed in the following 12 months of “regular” follow-up. Taken together, data from Yılmaz et al. confirm a favorable outcome of CML patients during COVID-19 pandemic, although their findings need confirmation in larger number and in an extended follow-up time.

Astonishing advances improved outcomes for CML patients over the last two decades, with their overall survival now approaching that of the general population. However, many research questions remain, and a significant minority of patients still face challenges. Issues such as managing long-term toxicity of TKIs, comprehensive care of the pediatric patient, optimizing monitoring in unexpected situations, and implications of the recent COVID-19 pandemic all stress the importance of expert physicians. We believe that the articles included in this Research Topic of *Frontiers in Oncology* will greatly assist hematologists in optimizing care for their CML patients.

## Author contributions

All authors listed have made a substantial, direct, and intellectual contribution to the work and approved it for publication.
